# Endogenous Pancreatic β Cell Regeneration: A Potential Strategy for the Recovery of β Cell Deficiency in Diabetes

**DOI:** 10.3389/fendo.2019.00101

**Published:** 2019-02-20

**Authors:** Fan Zhong, Yan Jiang

**Affiliations:** ^1^Department of Gastroenterology, Songjiang Hospital Affiliated First People's Hospital, Shanghai Jiao Tong University, Shanghai, China; ^2^Institutes of Biomedical Sciences of Shanghai Medical College, Fudan University, Shanghai, China

**Keywords:** pancreatic β cells, endogenous regeneration, pharmaceutical stimuli, rodent model, diabetes

## Abstract

Endogenous pancreatic β cell regeneration is a potential strategy for β cell expansion or neogenesis to treat diabetes. Regeneration can occur through stimulation of existing β cell replication or conversion of other pancreatic cells into β cells. Recently, various strategies and approaches for stimulation of endogenous β cell regeneration have been evaluated, but they were not suitable for clinical application. In this paper, we comprehensively review these strategies, and further discuss various factors involved in regulation of β cell regeneration under physiological or pathological conditions, such as mediators, transcription factors, signaling pathways, and potential pharmaceutical drugs. Furthermore, we discuss possible reasons for the failure of regenerative medicines in clinical trials, and possible strategies for improving β cell regeneration. As β cell heterogeneity and plasticity determines their function and environmental adaptability, we focus on β cell subtype markers and discuss the importance of research evaluating the characteristics of new β cells. In addition, based on the autoimmunologic features of type 1 diabetes, *NOD/Lt-SCID-IL2rg*^*null*^ (NSG) mice grafted with human immune cells and β cells are recommended for use in evaluation of antidiabetic regenerative medicines. This review will further understand current advances in endogenous β cell regeneration, and provide potential new strategies for the treatment of diabetes focused on cell therapy.

## Introduction

The pancreas plays an essential role in energy consumption and metabolism. It consists of two functionally and morphologically distinct components: the exocrine and endocrine. The exocrine pancreas is composed of acinar and ductal cells that secrete digestive enzymes. The endocrine pancreas is composed of five different hormone-secreting cell types that include glucagon-secreting α cells, insulin-producing β cells, somatostatin-releasing δ cells, ghrelin-releasing ε cells, and pancreatic polypeptide (PP)-secreting cells. These cells aggregate to form the islets of Langerhans, which are intermingled with the intra-islet microvascular network and play an essential role in regulation of blood glucose levels by directly secreting insulin and glucagon into the bloodstream. Type 1 diabetes (T1D) and type 2 diabetes (T2D) are defined as blood hyperglycemia caused by an absolute or relative deficiency of pancreatic β cells. Autopsy studies have shown deficits in β cell mass in approximately 70~100 and 0~65% in patients with T1D and T2D, respectively (1, 2). Therefore, β cell mass regeneration is a potential therapeutic strategy for recovery of β cell loss in patients with diabetes. Regeneration of β cells occurs through endogenous regeneration or exogenous supplementation, such as transplantation of cadaveric islets or grafting of new β cells generated from *in vitro* cell engineering. Recently, numerous strategies and technologies for producing human insulin-secreting cells have emerged, including *in vivo* stimulation of existing β cell replication, reprogramming of other pancreatic cells to differentiate into β cells, *in vitro* differentiation of induced pluripotential stem (iPS) cells into new β cells, and generation of human islets from genetically engineered pigs (3, 4). However, clinical application has remained a challenge. For example, strategies for enhancing replication of residual β cells have been successful in rodent but not in humans. In addition, drugs that stimulated conversion of α cells into β cells in animal experiments did not do so in clinical trials. As such, it is critical to determine the causes for limited success of clinical trials, and to determine possible strategies for improving cell therapy for T1D. In this review, we summarize advanced strategies and approaches for endogenous β cell regeneration, discuss regenerative mechanisms under physiological and pathological conditions, focus on various factors involved in stimulation of regeneration, and discuss promising potential pharmaceutical drugs. Moreover, as T1D is characterized by autoimmune-mediated β cells death, and heterogeneity and plasticity of β cells determine their function and environmental adaptability, we believe that thorough understanding associations between neogenetic β cells and diabetogenic autoimmune cells can lead to strategies to enhance the immunologic tolerance of neogenetic β cells, thus improving T1D cell therapy. In this review we introduce β cell subtyping markers that correspond with their functional features, and highlight the importance of using the humanized diabetic mice grafted with autoimmune cells and β cells in future studies.

## Replication of Existing Pancreatic β Cells

Pancreatic β cells replicate readily in the fetal and neonatal stages. However, this ability to replicate rapidly declines after these stages. Furthermore, this ability to replicate is different in rodents and humans. Proliferation of β cells is precisely controlled by cell cycle regulators and circulating soluble factors. Studies have shown that many mitogenic agents could stimulate β cell replication in young rodents, but not in humans. However, using high-throughput chemical screening, a series of inhibitors of DYRK1A-NFAT, GSK3, and NF-κB signaling pathways were shown to increase human pancreatic β cell replication, suggesting that these inhibitors have unique potential for treatment of diabetes.

### Replicative Ability of β Cells Over the Lifetime

During embryonic development, insulin-positive β cells appear at approximately embryonic day 13.5 in mice or during weeks 8–9 in humans. During the fetal period, β cells are mainly generated by differentiation of endocrine progenitor cells (5). During the late gestational and neonatal stages, β cells are generated by replication of existing β cells (6, 7). The rate of β cell replication reduces after weaning, and the renewal capacity of β cells becomes limited during adulthood or late adolescence. Nevertheless, β cell mass, which is determined on the basis of cell numbers and individual cell volumes, correlates in a linear fashion with body weight throughout the lifespan of an organism (5, 8). For example, in rats, the number and size of β cells expands with body weight during the first few months of life. The rate of β cell replication then progressively declines, to 1% in young rats (1 month of age), and <0.2% in adults (3~7 months) (8). In aging rats (15~20 months), β cell mass primarily increases through increased cell size (9). In healthy rodents, individual β cells have long lifespans, and replication of mature β cells is limited during adulthood (5, 10). Under some physiological or pathological conditions, rates of β cell proliferation are elevated. For example, β cells proliferate adaptively in response to pregnancy or obesity via self-replication (11–14). Moreover, in young rodents, β cell proliferation can be induced by increased metabolic demands or β cell deficiency resulting from tissue injury (8, 15).

### Different β Cell Replicative Ability Between Rodent and Human

Human and rodent islets have distinct structural and molecular characteristics (16). Replicative ability of human and rodent β cells have common and different features. For example, β cell mass increases during the earlier stages of life and declines with aging in both species. Adaptive β cell proliferation during pregnancy and obesity occurs extensively in rodents, but is limited in humans (17). Pregnancy-associated insulin resistance induces amplified insulin production to maintain glucose homeostasis. In rodents, elevated insulin production is accompanied by increased β cell numbers mediated by lactotrophic hormones (13, 14, 18). Humans also exhibit a compensatory increase in insulin secretion. New β cells originate from other pancreatic cell lineages and existing β-cells. Moreover, β cell proliferation mediated by lactotrophic hormones or other mitogenic stimuli is limited in humans (19). In addition, obesity-induced insulin resistance is associated with dramatic expansion of β cell mass in several rodent models (20), but not in human islets (20). Various mitogenic agents, hormones, and growth factors (GFs) such as Glp-1, Gip-1, exendin-4, prolactin, Hgf, and Igf-1 stimulate β cell proliferation in rodents but not in humans (21–27).

### Mediators of β Cell Replication

#### Cell Cycle Regulators

β cell replication is mediated by multiple mitogenic signaling pathways such as Irs–Pi3k–Akt, Gsk3, mTor, ChREBP/cMyc, Ras/Raf/Erk, and Nfats. These mechanisms also involve upstream activators of mitogenic signaling pathways, including nutrients (glucose, calcium), epidermal and platelet-derived GFs (Glp1, Gip), and hormones (leptin, estrogen, prolactin, and progesterone). Mitogenic signals stimulate quiescent β cells to re-enter the cell cycle by regulating the expression of downstream cell cycle regulators such as cyclins, cyclin-dependent kinases (Cdks), cell-cycle inhibitors, and E2F factors (28–33). For example, exendin-4 and glucagon-like peptide 1 (Glp-1) exert mitogenic effects on β cell proliferation by activating cell cycle activators (cyclin A and Cdk1) and proliferation-activating transcription factors (TFs) through the cAMP-dependent calcineurin/Nfat pathway (24, 25, 34–37). Menin is an endocrine tumor suppressor that suppresses β cell proliferation by epigenetically promoting the expression of the cell-cycle inhibitors p27 and p18 or by inhibiting K-Ras signaling (38–40). Moreover, Ezh2 mediates increased trimethylation of p16INK4a and p19Arf by H3K27, which epigenetically represses Ink4a/Arf production and contributes to proliferation of pancreatic β cells (41).

#### Circulatory Regulators

Circulating soluble factors derived from other organs act as systemic regulators that control β cell proliferation during puberty, pregnancy, and obesity. Multiple circulatory regulators have been implicated in control of β cell proliferation in response to insulin resistance. Examples of circulating regulators include intestinal peptides such as Glp-1 and Gip-1 (24, 25), adipose-tissue-derived adipokines such as adipsin (42, 43), resistin, and leptin (44, 45), and skeletal-muscle-secreted factors such as Il6 and Il10 (46, 47). In addition, crosstalk between hepatic and pancreatic tissues modulates β cell growth in response to insulin resistance. Many hepatocyte-derived factors have been identified as stimulators of β cell proliferation in mice and humans (48). In an insulin receptor knockout mouse model of insulin resistance, hepatocyte-derived secretory SerpinB1, and its partial mimic GW311616A, enhanced β cell proliferation by inhibiting elastase activity and activating key proteins in GF signaling (49). In addition, in mouse models of diabetes, exogenic expression of hepatic GFs, such as Hgf, Igf1, and Igf2, can regulate β cell mass by increasing β cell replication (50–53).

### Strategies for the Stimulation of β Cell Replication

Multiple approaches have been evaluated to rapidly and robustly replenish β cell masses. Numerous stimuli that promote β cell proliferation have been identified (38, 54–56). Studies have shown that administration of exogenous stimuli can stimulate β cell proliferation in young rodents. Whether adult rodent β cells can be induced to proliferate by exogenous stimuli remains unclear. Some studies have suggested that the replication of existing β cells induced by pancreatectomy (Px) or β cell apoptosis is the major source of new insulin-expressing cells in adult mice (6, 57). Other studies have indicated that various diabetogenic injuries, including partial Px, streptozotocin administration, and pancreatic duct ligation (PDL), cannot stimulate β cell proliferation in adult mice (21, 58, 59). Recently, high-throughput chemical screening has identified multiple potential agents for stimulation of β cell replication (60). As shown in Table 1, these agents include DYRK1A inhibitors (harmine, aminopyrazine compounds, and 5-iodotubercidin), which increase β cell proliferation by inhibiting calcineurin/Nfat/Dyrk1a signaling (61, 62, 64, 66). Osteoprotegerin and denosumab stimulate human β cell proliferation through inhibition of the receptor activator of NF-κB ligand pathway (63). Moreover, high-throughput RNAi screening has demonstrated that CDKN2C/p18 or CDKN1A/p21 silencing facilitated cell-cycle re-entry of quiescent adult human β cells (65).

**Table 1:** Potential drugs for increasing pancreatic β cell replication.

**Inhibitor**	**Rodent model**	**Human islet**	**Pathway**	**References**
Harmine	Px	h-islet-NSG	DYRK1A	(61)
5-iodotubercidin	ND	h-islet-NSG	DYRK1A	(62)
Osteoprotegrin	STZ-mice	β cells *in vitro*	RANKL, GSK3	(63)
Denosumab	ND	h-islet-NSG	RANKL	(63)
Aminopyrazine (GNF7156, GNF4877)	DTA-induced diabetic mice	h-islet-NSG	DYRK1A, GSK3B	(64)
Serpin B1 (sivelestat, W311616A)	C57Bl/6	h-islet-NSG	protease	(49)
shRNA targeting to p21 and p18	ND	Human islets *in vitro*	p18, p21	(65)

*ND, not determined; h-islet-NSG, human islet graphed into NSG mice*.

## Reprogramming of Other Pancreatic Cells Into β Cells

During embryonic development, pancreatic β cells are generated from multipotent pancreatic progenitors in a sequential and gradual process that is elaborately controlled by defined transcription factors. Pancreatic ductal epithelium cells and Ngn3 positive pancreatic cells are generally considered progenitors of β cells. They differentiated into β cells when islets were destroyed in rodent models. In this section, we introduce advanced strategies for conversion of non-β cells to β cells through ectopic expression of specific TFs or by pharmaceutical stimuli.

### TFs Regulate the Differentiation of Endocrine Cells

**Figure 1:**
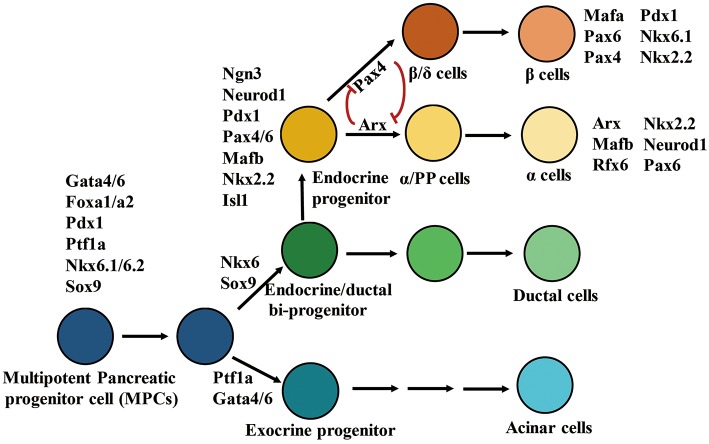
Transcription factors involve in differentiation of pancreatic cell lineages. In the diagrammatic sketch for pancreatic cell differentiation, the process of differentiation from multipotent pancreatic progenitors to α or β cells is presented; and the combination of key TFs determining the specialization of endocrine and exocrine pancreatic cell lineages is displayed. The figure was reproduced with permission from Elsevier and Copyright Clearance Center. This figure was adapted from Hang and Stein (67).

Development of embryonic pancreatic β cells is elaborately controlled by TFs involved in pancreatic determination (67) (Figure 1). During early pancreatic bud outgrowth, maintenance and specialization of multipotent pancreatic progenitor cells (MPCs) is modulated by the pancreatic TFs Gata4/6, Foxa1/2, Pdx1, Ptf1a, Mnx1, Sox9, Nkx6.1, and Hnf1β. Depletion of any of these TFs impairs pancreatic bud formation (68–72). Although Pdx1 is extensively expressed in pancreatic cells, it is only highly expressed in adult β cells. Lineage tracing studies in mice have demonstrated that Pdx1-positive MPCs possess high proliferative ability and can differentiate into all cell types in each of the three major pancreatic compartments (exocrine, endocrine, and ductal) (73, 74). Furthermore, the ectopic expression of Pdx1 or Ptf1a in the endoderm can induce ectopic pancreatic bud formation (71, 75). Ptf1a^+^/Gata4^+^ MPCs differentiate into exocrine progenitors, whereas Sox9^+^/Ndx6.1^+^ MPCs differentiate into endocrine/ductal bipotent progenitors (69, 76–79). Early endocrine progenitors originate from bipotent trunk duct-endocrine progenitors, and their differentiation is initiated by the expression of neurogenin 3 (Ngn3). Ngn3-deficient mice fail to generate endocrine cells, but undergo duct enlargement, and ectopic expression of Ngn3 directs pancreatic progenitors toward an endocrine fate. These phenomena suggest that Ngn3 is necessary for differentiation of islet cells (80–82). Endocrine development requires participation of other endocrine progenitor TFs such as Isl1, Neurod1, Pax6, Mafb, Nkx2.2, and Rfx6. These TFs are activated by Ngn3 and are involved in differentiation of endocrine cell lineages (80, 83). Finally, monohormonal islet cell lineages differentiated from Ngn3-positive endocrine progenitors are also regulated by a specific combination of TFs. For example, Pax4 and Arx participate in islet cell specialization via cross-inhibitory interactions. Pax4 and Arx promote the differentiation of islet progenitor cells into β/δ or α/PP cells, respectively (84). The α cell positive TF profile includes Arx, Mafb, Rfx6, Nkx2.2, Neurod1, and Pax6, whereas the β cell positive TF profile includes Nkx2.2, Pax4/6, Pdx1, Nkx6.1, and Mafα.

### Differentiation of Pancreatic Progenitors Into β Cells

Increasing β cell proliferation is a possible approach for recovery of β cell deficiency in diabetes. Stimulation of β cell neogenesis, however, may be a more feasible approach for treatment of diabetes than elevation of β cell proliferation given the nearly complete loss of β cells in T1D. Neogenesis is defined as formation of insulin-producing β cells through differentiation from stem/progenitor cells or conversion from other pancreatic cells. The existence of adult β cell progenitors remains the most controversial topic in diabetes research despite evidence showing that pancreatic cell lineages including ductal, endocrine, and exocrine, are derived from embryonic multipotent progenitors. Several studies have shown that β cells develop from other progenitor cells. The pancreatic ductal epithelium is a potential progenitor of islet and acinar tissues after birth (85). Foci of regeneration induced by partial Px comprise new ductal cells that express markers of embryonic pancreatic epithelium, including Pdx1, Hnf6, Foxa2, Tcf1/2, and Sox9, resulting in formation of new pancreatic lobes. These behaviors suggest that new ductal cells act as progenitors for the regenerating pancreas (86). In adult rodents, ductal cells differentiate into β cells in response to specific stimuli. For example, in adult mice Sox9 positive ductal cells differentiate into β cells in response to moderate hyperglycemia combined with long-term administration of low dose epidermal GFs (69, 87). In the PDL mouse model, pancreatic duct cells positive for the expression of carbonic anhydrase II, a duct cell-specific marker, act as progenitors of new islets and exocrine cells after injury (88–90). Recently, a selective Cdk5 inhibitor was identified that could promote β cell differentiation from ductal progenitors in zebrafish. This effect was observed in adult mice treated with PDL and human induced pluripotent stem (iPS) cells, which indicated that Cdk5 acts as an endogenous suppressor for β-cell differentiation (91). Moreover, in injured adult mouse pancreases, Ngn3-positive pancreatic cells act as endocrine progenitors and give rise to all islet cell types, including glucose-responsive β cells through the notch signaling pathway (73, 92, 93).

### Conversion of Other Pancreatic Cells to β Cells

β cell regeneration through transdifferentiation from other pancreatic cells, including exocrine and endocrine pancreatic cells, has been well-characterized. Results of genetic lineage tracing studies involving mouse models of severe β cell ablation have suggested that new insulin-producing β cells are generated from conversion of pancreatic α or δ cells in adult and adolescent mice (94, 95). Moreover, in diabetic mice transient treatment with epidermal growth and ciliary neurotrophic factors induces reprogramming of acinar cells to β cell masses (96). Differentiation of pancreatic lineages is sequentially and regionally regulated by pancreatic TFs (97). In adult mice, re-expression of the specific combination of Ngn3, Pdx1, and Mafα by adenoviral transduction contributes to reprogramming of pancreatic exocrine cells into insulin-expressing cells that are similar to β cells (98). Similarly, ectopic overexpression of β cell-specific TFs also induces reprogramming of exocrine or endocrine cells into β cells. In adult mice, ectopic expression of β cell-specific single TFs such as Pax1 or Pax4, ectopic expression of a combination of Pax1 and Mafα, or targeted disruption of α cell-specific TFs such as Dnmt1 and Arx, can induce conversion of α cells to β cells (74, 99–102). In addition, forced expression of Pax4 also mediates transdifferentiation of δ cells, Ngn3 positive endocrine progenitors, and duct-lining precursor cells into β cells (74, 103, 104).

### Potential Drugs for Stimulation of β Cells Conversion

Although ectopic expression of TFs effectively induces conversion of other pancreatic cell into β cells in mice, viral or transgene-mediated overexpression may be difficult to achieve in humans. As such, drug-stimulated conversion may be a potential alternative approach for T1D treatment. Recently, several small molecules have been identified as activators of β cell neogenesis.

#### γ-Aminobutyric Acid

γ-Aminobutyric acid (GABA), an inhibitory neurotransmitter in the central nervous system, is synthesized from glutamate by glutamate decarboxylase (GAD) (105). High levels of GABA and GAD are present in pancreatic islet cells, particularly β cells (106, 107). GAD65, an isoform of GAD, acts as a major autoantigen in T1D (108). GABA promotes β cell replication and inhibits β cell apoptosis in mouse models of STZ-induced diabetes and grafted human islets (109–112). GABA released from β cells interacts with and activates the ionotropic receptor GABA_A_ (a Cl^−^ ion channel) and the metabotropic G-protein-coupled receptor GABA_B_ in plasma membranes of islet cells (113–116). Binding of ligands to receptors enhance insulin secretion from β cells and suppress glucagon release from α cells (109, 117). Recently, Ben Othman reported that prolonged GABA exposure induced conversion of α cells into β cell-like cells in a mouse model of STZ-induced diabetes. Moreover, GABA treatment results in loss of α cells in grafted human islets and concomitantly increased islet mass and β cell-like counts. The mechanism of GABA-mediated conversion of α cells into β cells, however, requires further elucidation. The ability of GABA to downregulate Arx expression suggests that it acts on GABA_A_ receptors on α cells (118). In addition, GABA may act as an immunosuppressive regulator in T1D by mediating cytokine secretion from human peripheral blood mononuclear cells and CD4^+^ T cells (119, 120). In summary, administration of GABA contributes to replication of β cells, enhances conversion of α cells to β cells, and suppresses immune reactions in rodent models of diabetes. Given these actions, GABA has a potential antidiabetic role and clinical value for treatment of T1D.

#### Artemisinin

Artemisinin may act as a potential activator of conversion of α cells to β cells. One report showed that artemisinin impaired α cell identity and induce insulin expression in α cells through translocation of Arx from the nucleus to the cytoplasm, which then inhibited Arx. Moreover, the mechanism of action of artemisinin on transdifferentiation of α cells into β-like cells involves enhancement of GABA receptor signaling in a gephyrin-dependent manner (121). However, Meulen et al. reported that stimulation of intact islets with high doses of artemether failed to promote transdifferentiation of primary α cells to β cells. Moreover, artemisinin reduces *Ins2* gene expression, suppresses glucose uptake, and abrogates calcium responses and insulin secretion in response to glucose (122). These paradoxical effects of artemisinin on β cell regeneration warrant further verification.

#### Diet Therapy

Interestingly, a novel diet therapy with 4-day fasting-mimicking diet (FMD) cycles can reverse β cell failure and can reverse diabetes in mice. FMD promotes Ngn3-driven β cell regeneration by inducing re-expression of prenatal development genes, such as *Sox17* and *Pdx1*, in the adult pancreas (123).

In addition, the mechanism underlying maintenance of the correct proportion of cellular components in neogenesis of islets derived from conversion of non-β cells has yet to be determined. For example, how the pancreas compensates for loss of α cells during conversion of α cells to β cells requires further study. One potential mechanism is that α cell conversion includes mobilization of duct-lining precursor cells (reawakening of the epithelia-to-mesenchymal transition), regeneration of α cells, and conversion of α cells to β cells (118). This mechanism suggests that neogenetic β cells originate from neogenetic α cells that differentiated from mobilized Ngn3-positive endocrine progenitor cells. Accordingly, formation of new islets is a complex and dynamic process, and new islets may contain different cell types or even intermediate transitional cells. Tracking the fate of these cells through lineage tracing, and identifying them by single cell analysis would help reveal mechanisms of α cell conversion (124–126).

## Rodent Models For Study of β Cell Regeneration

**Figure 2:**
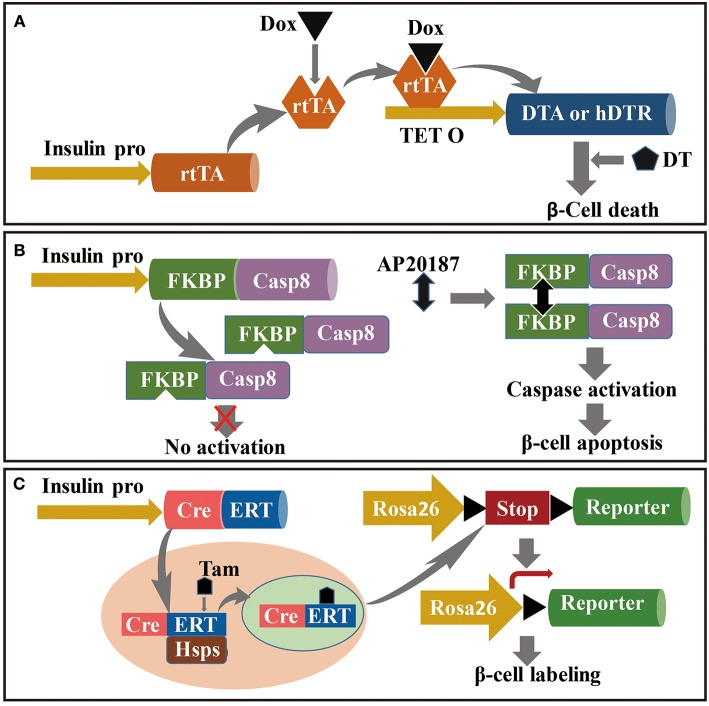
Mouse model for the study of β cell regeneration. The targeting to pancreatic β cells is relayed on the driving of insulin promoter, and conditional ablation (or labeling) is dependent on the induction under Dox, AP20187 or Tam administration in specific transgenic strains. **(A)** Dox-induced DT-dependent specific ablation of β cells; **(B)** Caspase-FKBP-induced apoptosis of pancreatic β cells conditionally activated by AP2018; and **(C)** Tam-induced lineage tracing of pancreatic β cells based on CreERT: Rosa26-LoxP-Reporter transgenic strains.

Diabetic rodent models, which generally include classical and genetic models, play important roles in the study of the molecular mechanisms of β cell regeneration and the evaluation of the effects of potential pharmaceutical drugs for diabetes treatment. Classical models are defined by damage of β cells by surgery or treatment with chemical compounds, such as Px, PDL, and STZ-mediated β cell ablation. Genetic models are constructed through crossing special transgenic mouse strains, which leads to specific and inducible β cell ablation or allows for tracking of target islet cells (Figure 2). In this section, we comprehensively introduce principles and protocols for construction of these rodent diabetic models, and review the recent advances in β cell regeneration through use of these models. In addition, based on autoimmunologic features of T1D and difficulties in stimulation of β cell regeneration in humans, we introduce a humanized diabetic mouse model, and suggest use of this model for study of β cell regeneration.

### Classical Diabetic Rodent Models

Classical rodent models of β cell regeneration include partial Px, PDL, and STZ-mediated β cell ablation. ***Px***: Removal of 60~90% of the adult rat pancreas through Px administration induces extensive pancreatic regeneration with formation of new lobes and islets, and proliferation of acinar cells. The Px model has been used to study β cell neogenesis and replication (86, 127, 128). ***PDL***: PDL is defined as surgical ligation of the pancreatic duct at the level of the pylorus. This procedure obstructs drainage of exocrine secretions and results in loss of acinar cells through death and dedifferentiation. During the early years of diabetes research, this model was widely used to study mechanisms of β cell formation (129–131). More recently, the PDL model has been used to demonstrate expansion of β cells generated from Ngn3-positive endogenous progenitors (92). A controversial finding, however, suggested that PDL failed to elevate β cell levels in mice (132, 133). ***STZ-mediated β cell ablation***: STZ, a cytotoxic chemical produced by *Streptomycetes achromogenes*, can be used to cause extensive damage to endogenous β cells and induce hyperglycemia. It binds to the Glut-2 transporter, which is abundantly distributed on plasma membranes of β cells. STZ causes DNA alkylation and generates high levels of free radicals that cause DNA damage and cell death. STZ can be used alone or in combination with other chemicals to induce diabetes. STZ-induced β cell damage causes spontaneous β cell regeneration in neonatal and adult rodents (134–136). STZ administration, however, has failed to stimulate an adaptive increase in β cells in adult monkeys (137). The STZ model has been used to study β cell regeneration induced by various stimuli, including transgenes and signaling pathway activators (52, 138–142).

### Genetic Modification Induces β Cell Ablation

#### Diphtheria Toxin-Targeted Specific and Inducible β Cell Ablation

*Corynebacterium diphtheriae* produces diphtheria toxin (DT) as a single secretory polypeptide. *In vitro*, mature DT generates two components, fragments A (DTA) and B (DTB). DT binds to a DT receptor on cell surfaces and is incorporated into cells through receptor-mediated endocytosis. DT can inactivate elongation factor (EF)-2 in cells by catalyzing transfer of ADP-ribose to EF-2. Inactivation of EF-2 inhibits protein synthesis and causes cell death (143). Thus, specific and inducible killing of pancreatic β cells is based on doxycycline (Dox)-induced expression of DT. The approach for construction of this model is crossing an insulin-reverse tetracycline-dependent transactivator (*insulin-rtTA*) transgenic mouse strain with the *TetO-DTA* mouse. RtTA expression in the *insulin-rtTA* mouse strain is driven by a rat insulin promoter, whereas DTA subunit expression in the *TetO-DTA* mouse strain is driven by a rtTA-responsive promoter. In the Tet–on system, rtTA interacts with the tet-resistance operon in the presence of Dox and activates transcription (Figure 2A) (144). Accordingly, administration of Dox to double-transgenic mice induces DTA expression in β cells, resulting in widespread β cell apoptosis (15). Moreover, DT binding affinity for human DT receptors is 10^5^-fold higher than that of murine DT receptors (145). Conditional and targeted cell ablation in mice can be achieved through transgenic human DTR expression driven by specific promoters in the presence of DT. Numerous studies have documented that ectopic expression of human DTR driven by insulin or glucagon promoters resulted in targeted ablation of 99% of α or β cells following DT administration (94, 95, 146).

#### Caspase 8–FKBP Transgene Induces β Cell Apoptosis

In this section, mechanisms of caspase-FKBP-induced specific-cell ablation are summarized in context of conditional caspase activation driven by cell-specific promoters. Using the *caspase-FKBP* transgenic model, multiple tissue cells have undergone conditional and specific ablation such as cardiac myocytes, adipocytes, hepatocytes, and pancreatic β cells (147–151). The caspase-FKBP fusion protein was designed with human caspase catalytic domains such as p20 and p10 from caspase 8 fused with a series of FKBPv domains such as Phe36Val mutant FKBP. The binding affinity of the caspase–FKBP fusion protein for the FK506 analog AP20187 is 1,000-fold greater than that of endogenous FKBP. The mechanism underlying caspase activation through the forced dimerization of adjacent FKBP molecules by AP20187 is shown in Figure 2B (148–150). Accordingly, a PANIC–ATTAC mouse model was constructed through transgenic expression of FKBPv–caspase 8 fusion protein driven by a rat insulin promoter. This model has been used to study inducible and reversible β cell ablation and other aspects of diabetes (150, 151).

### Genetic Cell Lineage Tracing Model

Genetic cell lineage tracing has been used for tracking target cells in the body, and enables visualization of the source and fate of target cells. This approach has been used to analyze formation and regeneration of β cells under physiological or pathological conditions (152). For the past several decades, the *Cre/loxP* genetic lineage tracing system has been the most widely used method for tracking cell fate (153). The *Cre/loxP* system functions through expression of *Cre* recombinase driven by the *loxP-stop-loxP* cassette, a cell-specific promoter located upstream of the reporter gene. Cre recombinase excises the floxed STOP cassette and subsequently activates reporter gene expression. Conditional or inducible expression of Cre recombinase is based on the *TetO-Cre* or *CreER*^*TM*^ transgenic mouse strains, which are crossed with the *ROSA26*/reporter mouse strain and result in inducible tracking for target cells. The *CreER*^*TM*^ transgenic cassette contains a fusion of *Cre* recombinase with a mutated ligand-binding domain (*ER*^*TM*^), the latter of which preferentially binds to the antiestrogen tamoxifen instead of endogenous 17β-estradiol (154). Under normal conditions, the CreERT fusion protein is sequestered by HSPs in the cytoplasm. Following treatment with tamoxifen, tamoxifen binds to CreERT, resulting in disruption of the interaction with Hsp90. Released CreERT transfers to the nucleus, initiates recombination, and activates reporter gene expression (Figure 2C) (155, 156). Numerous experiments aimed at identifying the source or fate of pancreatic cells in rodent models have been performed using the conditional *Cre-LoxP* genetic lineage tracing system (6, 103, 104, 123). For example, Fabrizio crossed *RIP-DTR, RIP-CreER*^*TM*^, and *ROSA26-LoxP-YFP* transgenic mouse strains to achieve DT-dependent conditional β cell ablation and tamoxifen-dependent β cell lineage tracing. Moreover, hybridization among the transgenic mouse strains *Glucagon-rtTA, TetO-Cre*, and *ROSA26-LoxP-YFP* was used to track the source of insulin^+^ cells. This study found that 65% of insulin-expressing cells following β cell ablation were YFP^+^, which indicated that neogenetic insulin-expressing cells originated from α cells (94).

### Humanized Diabetic Mice Model

T1D is an autoimmune disease caused by immune-mediated destruction of pancreatic β cells. The etiology of T1D involves interactions among genetic, environmental, and immune factors. Multiple approaches for prevention or treatment of T1D have been have been successfully used in the non-obese diabetic (NOD) mouse model, but have not been successfully reproduced in humans. These failures may be attributed to structural and compositional variations between NOD murine and human islet cells, as well as to differences between human and murine immune systems (157, 158). These differences include genetic susceptibility loci, immune responses to environmental factors, leukocyte subsets, and immunological factor compositions (159, 160). To define the interaction between the human immune system and β cells, and to improve treatments for T1D, a humanized diabetes mouse model in which mice are grafted with a functional human immune system and human β cells closely imitates the physiological conditions of human T1D. The immunodeficient *NOD/Lt-SCID-IL2rg*^*null*^ (NSG) genotype constructed on NOD mice is suitable for grafting of human immune cells and human β cells because it contains genetic modifications of severe combined immunodeficiency (*SCID*) mutation and a complete null mutation of the *IL2rg* gene (160–163). NSG mice can be grafted with human tissue, hematopoietic stem cells, and peripheral blood mononuclear cells (162, 164). Recently, studies of immunological mechanisms of T1D, treatment strategies for diabetes, and transplantation of islet cells using NSG mice have made significant progress (165–170). Thus, this model will be a powerful platform for finding potential drug targets for T1D therapy and evaluating antidiabetic drugs in preclinical trials.

## Characterizing Neogenetic Islet Cells

New β cells are typically described as β cell-like cells, which often possess the basic characteristics of β cells such as glucose-stimulated insulin secretion. However, β cells are heterogeneous and exhibit plasticity during development, under pathological conditions, or following specific treatments. Therefore, it is necessary to identify characteristics and subtypes of new β cells.

### Features of Mature β Cells

Embryonic and neonatal β cells are immature and can produce insulin, but lack the ability to respond to glucose stimulation. Within days after birth, β cells develop the ability to secrete insulin in response to glucose (glucose-stimulated insulin secretion; GSIS) and become mature functional β cells (171). Mafa, NeuroD, and Errγ drive β cell maturation, and urocorin3 (Ucn3) acts as a marker of β cell maturation (172–176). Immature β cells that lack Ucn3 are present throughout life. These cells are involved in the intermediate stage of transdifferentiation of α cells into β cells and may be potential sources for β cell regeneration (177). Therefore, given that the maturation state of β cells affects insulin production, secretion, and GSIS, subtyping new β cells will allow for better understanding of β cell characteristics and functions.

### New Markers for Subtyping of β Cells

Under pathological conditions or in response to specific treatments, adult β cells exhibit heterogeneous responses (178). During the past few decades, a series of markers of β cell heterogeneity have been identified and characterized (Table 2). For example, insulin and Pdx1 levels reflect different maturation states of β cell subpopulations (179, 185). Expression of Glut2^lower^ characterizes rare subpopulations of β cells with low insulin content, properties of stem/progenitor cells, and lineage plasticity that appears during β cell regeneration induced by β cell ablation (180). The correlation between E-cadherin and insulin levels in adult rodent β cells suggests the importance of tight cell-to-cell junctions to the function of β cells (181, 186). Recently, novel proteins have been identified as phenotypic and functional markers for discrimination of β cell populations (187). Flattop, a Wnt/planar cell polarity effector, can be used to distinguish the proliferative competence of mature β cells. Subpopulations of β cells labeled by Flattop present distinct molecular (gene expression level of Ucn3 and MafA), physiological (expansive ability to respond to stimulation), and ultrastructural features (182, 188, 189). Other subtyping markers, St8sia1 and Cd9, based on their expression levels, human β cells have been divided into four subtypes, which have diverse gene expression profiles and distinct basal and GSIS ability (183). In addition, hubs are the markers for β cell subpopulations with transcriptional immaturity and high metabolism. Hubs were discovered through studies of optogenetics and photopharmacology (184).

**Table 2:** The markers for subtyping of pancreatic β cells.

**Markers**	**Characteristics of β cells**	**References**
Ucn3, insulin, Pdx1	Mature state	(176, 179)
Glut2	Insulin secreting	(180)
E-cadherin	Insulin levels	(181)
Flattop	Proliferation-competent	(182)
St8sial and Cd9	Basal or glucose-stimulated insulin secretion	(183)
Hubs	Insulin secretion and glucose-response	(184)

Functional cooperation among islet cells is dependent on the three-dimensional architecture and cellular composition of islet cells (190). β cell subpopulations combine with other islet cells to form a three-dimensional islet architecture that contributes to distinct functions and influences development of diabetes mellitus. In addition, the physiological and pathological conditions of the pancreas also affect β cell heterogeneity (191, 192). In this process, β cells adapt physiologically, morphologically, and functionally to specific environmental cues. Therefore, given that heterogeneity and plasticity of β cells determine their functional and environmental adaptability, subtyping new insulin expressing cells would provide new clues for treatment of diabetes.

## Discussion

Over the past several decades, studies examining endogenous β cell regeneration have proposed numerous strategies for treatment of β cell-deficient diabetes. Most of these strategies, however, have only been successfully applied to animals. Although some treatment strategies for diabetes have been successful in rodent models, most have failed in humans. It is generally accepted that the autoimmunologic features of T1D are the primary causes of clinical failures. Specifically, neogenetic β cells are always recognized and attacked by diabetogenic T cells, which results in death of these new β cells. Fortunately, the approaches and technologies for protection of new β cells in islet transplantation have improved. For example, encapsulating technologies protect grafted islets from the host immune system (193, 194). Another strategy is to improve immunologic tolerance of new β cells through replenishment of regulatory T cells (195–198). Clinical trial results showed that prolonged immunosuppression in chronic T1D patients slightly increased native pancreatic insulin production, which demonstrated the effect of the immune system on endogenous pancreatic β cell regeneration (199). Moreover, technologies for generation of insulin producing cells derived from iPS cells has allowed for autologous β cell transplantation for T1D treatment (200, 201). However, because T1D is an autoimmune disease, new β cells are attacked by immune cells. Thus, further studies should focus on increasing the autoimmunologic tolerance of new β cells. First, we suggest focusing on the functionality and immunogenicity of new pancreatic β cells to improve adaptability in clinical applications. Subsequently, steps should be taken to improve understanding of the characteristics of pancreatic islets, islet cells, and new insulin-expressing cells. Furthermore, studies aimed at determining molecular mechanisms and potential regenerative medicines should use humanized mouse models of diabetes, which will provide significant information for development of new T1D therapies.

## Author Contributions

YJ designed and wrote the paper. FZ collected the data.

### Conflict of Interest Statement

The authors declare that the research was conducted in the absence of any commercial or financial relationships that could be construed as a potential conflict of interest.
